# Behavior and Immune Response of Conventional and Slow-Growing Broilers to *Salmonella* Typhimurium

**DOI:** 10.3389/fphys.2022.890848

**Published:** 2022-05-02

**Authors:** Ashlyn M. Snyder, Sean P. Riley, Cara I. Robison, Darrin M. Karcher, Carmen L. Wickware, Timothy A. Johnson, Shawna L. Weimer

**Affiliations:** ^1^ Department of Animal and Avian Sciences, University of Maryland, College Park, MD, United States; ^2^ Department of Veterinary Medicine, University of Maryland-College Park, College Park, MD, United States; ^3^ Virginia-Maryland College of Veterinary Medicine, College Park, MD, United States; ^4^ Department of Animal Science, Michigan State University, East Lansing, MI, United States; ^5^ Department of Animal Sciences, Purdue University, West Lafayette, IN, United States; ^6^ Department of Poultry Science, University of Arkansas, Fayetteville, AR, United States

**Keywords:** broiler, growth rate, *Salmonella*, immune response, behavior

## Abstract

Fast growth rate in broiler chickens comes with welfare concerns and the contribution of growth rate to pathogen resistance and sickness behavior is relatively unknown. The objective of this study was to evaluate physiological and behavioral responses of conventional (CONV) and slow-growing (SG) male broilers challenged with *Salmonella* Typhimurium. CONV (*n* = 156) and SG (*n* = 156) chicks were raised in a pen with wood litter shavings until day 7 of age, when birds were transferred to 24 isolators (*n* = 11 chicks/isolator). On day 14 of age, half of the birds (*n* = 12 isolators) were challenged with *S.* Typhimurium (ST) and the other half (*n* = 12 isolators) received a control (C). On days 7, 13, 17, 21, and 24, body weight was recorded, and blood, jejunum and ileum sections were collected from 2 birds/isolator (*n* = 48 birds/sampling) to measure plasma IgA and IgG and intestinal histomorphology, respectively. On days 12, 16, 21, and 23, video was recorded to evaluate bird postures (sitting, standing, or locomoting) and behaviors (eating, drinking, preening, stretching, sham foraging, allopreening, and aggression). CONV birds were 70 g heavier (*p =* 0.03) on day 21 and 140 g heavier (*p* = 0.007) on day 24 than SG. On day 7, CONV jejunum villus height and crypt depth were 22 and 7 μm greater (*p* ≤ 0.001), respectively, than SG. On day 24, ST ileum villus height was 95 μm shorter (*p* = 0.009) than C. IgA increased after day 17 for all birds and at day 21, CONV IgA was greater (*p* = 0.01) than SG. Although SG IgG was 344 μg/ml greater (*p* = 0.05) than CONV on day 7, CONV IgG increased with age (*p* < 0.0001) to greater (*p* ≤ 0.03) concentrations than SG on day 21 and day 24 by 689 μg/ml and 1,474 μg/ml, respectively, while SG IgG remained at similar concentrations after day 13. Generally, a greater proportion of birds sham foraged as they aged (*p* < 0.0001). A greater proportion of CONV tended to sit (*p* = 0.09) and fewer locomoted (*p <* 0.0001) than SG as they aged. The results illustrate conventional and slow-growing broilers differ in their behavior, immunity, and response to *Salmonella*.

## Introduction

The United States broiler industry is the largest globally, producing nearly 20 billion kg of chicken meat annually (NCC. National Chicken Council, 2021). As such, broilers are bred for increased feed efficiency and meat yield to satisfy consumer demand (Zuidhof et al., 2014), resulting in conventional broilers that reach market weight at 42 days of age (Aviagen, 2018). However, selection for fast growth comes with trade-offs, as breeds more intensively selected for growth rate have been observed to have greater mortality and culls (Dixon, 2020), greater incidences of hock burn (Weimer et al., 2020), greater disease susceptibility (Swinkels et al., 2007), and poorer immune function (Cheema et al., 2003). Thus, the welfare of conventional, fast-growing broilers has been called into question and compared against slow-growing broilers in recent years.

Torrey et al. (2021) studied the growth, efficiency, and mortality of sixteen broiler breeds to distinguish the outcomes of selection for growth. Body weight and feed efficiency varied, and the conventional breeds were up to 1,264 g heavier and were more feed efficient (by 43 points) than the other breeds, yet mortality did not differ amongst all breeds (Torrey et al., 2021). Other researchers have found greater plasma concentrations of enzymes (aspartate transaminase, creatine kinase, lactate dehydrogenase, and creatinine) and lower tibia ash in a conventional breed compared to four slow-growing breeds, indicating reduced liver function and poorer skeletal mineralization and support relative to body mass (Mohammadigheisar et al., 2020). Dixon (2020) compared three commercial broiler breeds to a slower-growing breed and reported poorer welfare measures (observational scores for gait, feather cover, feather cleanliness, and hock burn) and greater inactivity in the conventional breeds. Despite the current parameters studies have collected regarding conventional versus slow-growing broilers, little is known about the outcomes of the selection for growth rate on resistance to human foodborne pathogens, such as *Salmonella*.

*Salmonella enterica* serovars Enteritidis and Typhimurium are a common cause of human foodborne illness globally (Knodler and Elfenbein, 2020), resulting in 1.35 million infections and 420 deaths annually in the United States (CDC. Centers for Disease Control and Prevention, 2020). Contaminated poultry products, including chicken meat and eggs, are a frequent cause (Bearson et al., 2017). *Salmonella enterica* infection rarely causes clinical symptoms in poultry (Barrow et al., 2012), and chickens are reported to be asymptomatic carriers of *Salmonella* (Shanmugasundaram et al., 2019). However, subclinical physiological and behavioral indicators may exist as *Salmonella* infection can induce immune stress in chickens, impacting welfare and performance. This can include intestinal inflammation (Gomes et al., 2014), reduced appetite (Liu et al., 2014), and impaired gut morphology (Shao et al., 2013). Thus, the prevalence of *Salmonella* in broiler flocks and products is an important production, food safety, and human health concern, but little research has focused on the pathogen resistance of broiler breeds that differ in growth rate.

Both innate and adaptive immune responses are stimulated by *Salmonella enterica* infection within the chicken gastrointestinal system, involving both cytokine action (Brisbin, 2011; Crhanova et al., 2011) and antibody responses (Hassan et al., 1991; Holt et al., 1999; Acevado-Villanueva et al., 2020). Dar et al. (2019) orally challenged 4-day old Cobb broiler chicks with *S.* Typhimurium and reported elevated serum levels of immunoglobulins (Ig) M and IgG as well as increased mRNA expression of the cytokines interferon-gamma (IFN-γ), interleukin (IL)-12, and IL-18. Immune function differences exist between broiler breeds (Cheema et al., 2003; Swinkels et al., 2007). In a study evaluating the susceptibility to intra-abdominal *Salmonella* Enteritidis infection of two commercial broiler breeds and their crosses, Swaggerty et al. (2005) found that one parent breed and one crossbred breed had a greater number of heterophils at the infection site and increased mRNA cytokine expression. Immune function can also be selectively bred (Yunis et al., 2000; Cheng et al., 2004) and an inverse relationship has been observed between growth rate and immune function (Parmentier et al., 2010; van der Most et al., 2010). When chickens from a broiler breed and a layer breed were injected with lipopolysaccharide (LPS), the layers had greater mRNA expression of pro-inflammatory cytokines IFN- γ and interleukin 1 beta (IL-1β) compared with broilers (Leshchinsky and Klasing, 2001). Bodily resource reallocation in response to infection may be a primary contributor to the differences reported in the aforementioned research (Leshchinsky and Klasing, 2001; Parmentier et al., 2010; van der Most et al., 2010), because the intensive selection of birds for enhanced growth may prioritize the allocation of bodily resources towards growth as opposed to immune function (Humphrey and Klasing, 2004).

Selection for enhanced growth rate may also result in differences in gastrointestinal tract anatomy and nutrient absorption (Yamauchi et al., 2010). Yamauchi and Isshiki (1991) compared the intestinal structure of chickens from commercial broiler and layer breeds for 30 days post-hatch and reported that by day 10, the broilers had wider villi and microvilli as well as more epithelial cell turnover in the duodenum and jejunum. On the other hand, a recent study involving five broiler breeds varying in growth rate reported no differences in jejunum morphology (Mohammadigheisar et al., 2020). Infection of the gut by pathogens such as *Salmonella* Typhimurium (Kaiser et al., 2000; Dar et al., 2019) and *Salmonella* Enteritidis (Awad et al., 2016) can damage the intestinal epithelium, potentially impairing intestinal function. However, there is little research studying breed-related differences in gut integrity and resistance to pathogenic infection.

Differences in the behavior of fast- and slow-growing broilers have been previously studied (Almeida et al., 2012; Dixon, 2020; Torrey et al., 2020; Abeyesinghe et al., 2021). Generally, activity (locomoting and standing) decreases with age and conventional broilers are less active than slow-growing broilers (Dixon, 2020; Torrey et al., 2020). Additionally, conventional broilers tend to forage less than medium- (Almeida et al., 2012) and slow-growing (Yan et al., 2021). Exploratory behaviors (foraging), alongside comfort and social behaviors, such as stretching, preening and allopreening, can be studied to determine welfare status (Prayitno et al., 1997; Costa et al., 2012) and changes in these behaviors may precede clinical signs of disease (Abeyesinghe et al., 2021). This is particularly important because chickens (evolutionarily prey animals) rarely show sickness behavior when diseased.

Sickness behavior serves as an indicator of an immune response in action and is a sign of proinflammatory cytokine signaling (Kelley et al., 2003; Dantzer, 2004). There is a gap in knowledge regarding breed-related differences in the occurrence of sickness behaviors in broilers, especially their response to *Salmonella enterica* infection, but the selection for fast growth is reported to reduce proinflammatory cytokine expression (Leshchinsky and Klasing, 2001; Berghman, 2016). Thus, it is possible that broilers more intensively selected for fast growth rate may have reduced sickness behavior expression and otherwise display less significant changes in activity, exploratory, comfort, and social behaviors as a result of infection.

The use of conventional or slow-growing broilers is presently a hotly debated topic in animal welfare. At the same time, *Salmonella* infection in humans remains a continuous global concern, despite the rigorous industry efforts to control its spread. It is unclear if the selection for growth rate in broilers has impacted foodborne pathogen resistance. The objective of this study was to evaluate differences in body weight, immune response, gut morphology, and sickness behavior in conventional and slow-growing broiler chickens when challenged with *Salmonella* Typhimurium.

## Materials and Methods

### Experimental Design, Animals, and Housing

All procedures were approved by the University of Maryland Animal Care and Use Committee (IACUC#: R-NOV-19-55). The study was a 2 × 2 split plot design. Three-hundred and twelve male day-of-hatch chicks from two breeds, conventional (**CONV**; N = 156 Ross; Aviagen) and slow-growing (**SG**; *n* = 156 Redbro; Hubbard), were placed into a single floor pen with wood shavings litter. On day 7 of age, chicks were transferred to an ABSL2 research facility where 11 birds from each breed were exclusively placed into 6 isolators in each of 4 rooms (*n* = 24 isolators). Fresh water and commercially available feed (Purina Start and Grow Non-Medicated Chicken Feed) were provided *ad libitum* to all experimental treatment groups. Ambient temperature, humidity, and photoperiod were maintained according to the Ross Broiler Management Handbook (Aviagen, 2018) throughout the study.

A nalidixic acid (NAL)-resistant culture of *Salmonella enterica* serovar Typhimurium Strain #289-1 (Cox and Blankenship, 1975) was used as the challenge treatment. On day 14 of age, CONV (N = 6 isolators) and SG birds (*n* = 6 isolators) were challenged with 1 ml of 1.3 × 10^8^ CFU/ml in tryptic soy broth (TSB; **ST**) via oral gavage, while the controls received 1 ml sterile TSB (**C**).

### Qualitative Bacteriology for *Salmonella* spp.

Qualitative bacteriology was performed day of hatch (day 0) and 1 day prior to challenge (day 13) to ensure the birds were negative for *Salmonella* spp. prior to challenge. On day 0, 30 birds (*n* = 15 birds/breed) were randomly selected and their vents aseptically swabbed. Swabs were individually incubated in 10 ml TSB diluted with phosphate-buffered saline (PBS) for 24 h at 37°C. Swabs were then plated on bismuth sulfite agar (BSA) plates and incubated at 37°C for 24 h. Qualitative bacteriology for *Salmonella* spp. was repeated on day 13 with 48 birds (*n* = 24 birds/breed) using the same protocol as on day 0. Each swab from day 13 was plated on BSA as well as BSA + NAL plates to confirm birds were negative for *Salmonella* spp*.* and NAL-resistant *Salmonella* Typhimurium, respectively.

### Sampling and Video Recording

Mortality was recorded daily. Body weight, blood, and intestinal tissue samples were collected on days 7, 13, 17, 21, and 24 from 24 birds from each breed (*n* = 48 birds/sampling day). Birds were euthanized after recording live body weight, and blood was collected through cardiac puncture and was decanted into plasma separation tubes. Blood was centrifuged for 10 min at 2,000 × g and 15°C to separate plasma. A 2 cm longitudinal segment was removed from the jejunum (2 cm anterior to Meckel’s diverticulum) and the ileum (2 cm anterior to the ileocecal junction) and transferred to tubes containing 10% buffered formalin. On days 12, 16, 20, and 23, video was recorded on 2 isolators per room (*n* = 8 isolators total, 4 CONV and 4 SG) for 1 h using GoPro (GoPro Hero 7, San Mateo, CA) cameras. Days selected for recording video preceded each sampling by 24 h.

### Plasma IgA and IgG

Plasma was stored at −80°C until assayed with commercially available ELISA kits (Bethyl Laboratories Inc., Montgomery, TX) to determine immunoglobulin A (IgA) and immunoglobulin G (IgG) concentrations. The manufacturer protocol was followed using the recommended sample dilutions (1:1000 for IgA, and 1:100,000 for IgG). Absorbance was measured on a plate reader at 450 nm. A standard curve was generated for each plate to calculate sample IgA and IgG concentrations (μg/ml).

### Gut Morphology

Histological preparation was performed by Histoserv, Inc. (Germantown, MD). Slide images were taken at ×40 magnification and later measured using histological software (Qupath Quantitative Pathology & Bioimage Analysis, University of Edinburgh, United Kingdom). Five to ten villus and crypt measurements per intestinal segment (jejunum and ileum) per bird were recorded electronically for villus height and crypt depth. Villi were measured from the tip of the villus to the base at the villi-crypt junction, and crypts were measured from the villi-crypt junction to the crypt base at the basolateral membrane (Golder et al., 2011). Only well-oriented, untorn villi and their paired crypt were measured. Villus-crypt ratio (VCR) was calculated by dividing villus height by its corresponding crypt depth for each villus-crypt pair.

### Behavior

Each bird per video recording was coded for mobility, production, comfort, exploratory, and social behaviors by 3 individuals using instantaneous scan sampling every 15 s for the 60 min of video recorded (*n* = 241 scans/video), beginning 5 min after the researcher left the room. During each scan, the total number of birds performing each posture (sitting, standing, and locomoting), then each behavior (eating, drinking, preening, stretching, sham foraging, allopreening, and aggression) were recorded using the ethogram in Table 1. Each bird was coded for one posture and one behavior, one posture and no behavior, or not visible. Bird counts were calculated into proportions of birds performing a posture or behavior out of the total number of birds in the isolator for statistical analysis. Interobserver agreement was 95%.

**TABLE 1:** Ethogram of postures and behaviors.

Posture/Behavior^a^	Description
Sitting	Resting with hocks on the ground or lying on the side
Standing	One or both feet on the floor and immobile
Locomotion	Mobile and taking steps in any direction, at any speed of movement (walk or run). This may include jumping or lunging
Eating	Sitting or standing in front of the feeder with head over or in trough
Drinking	Sitting or standing next to the waterer and actively putting beak in the water or raising head to swallow
Preening	Sitting or standing and actively grooming oneself (body, feathers, feet, head) via pecking, running beak through feathers
Stretching	Standing while extending a leg and/or wing away from the body
Sham Foraging	Sitting, standing, or moving while physically investigating the environment by pecking or scratching
Allopreening	Sitting, standing, or moving while preening other birds
Aggression	Sitting, standing, or moving while interacting with another bird with vigorous pecking (usually directed at the recipient’s head) or kicking (physical contact). Also includes threats (no contact) with erect necks, raised neck feathers, and intentional movement with a raised head by the initiator to the recipient bird

a Ethogram adapted from Prayitno et al., 1997; Bizeray et al., 2002; Bokkers and Koene, 2003, Bailie et al., 2013, Baxter et al., 2019.

### Statistical Analysis

The bird was the experimental unit on day 7 and isolator was the experimental unit for subsequent ages. Due to collection errors at sampling, day 13, 17, and 21 histology data is missing, and histology results are only reported for days 7 and 24. Day 7 body weight, histology, and immune marker data were run in JMP (v14) using 1-way ANOVA for the fixed effect of breed. Day 13, 17, 21, and 24, body weight, histology (day 24 only), and immunoglobulin data were run using 2-way ANOVA for the fixed effects of breed, challenge, and their interaction with the random effect of isolator nested within room. Additionally, a model was run independent of challenge to evaluate the effects of age, breed, and their interaction with the random effect of isolated nested within room across all ages on broiler plasma immunoglobulin A and G (IgA and IgG) concentrations. Means were separated using Tukey’s post-hoc adjusted LSMeans. Pearson’s pairwise correlations were compared for body weight, immune markers, and histology data.

Behavior proportion data from days 12, 16, 20, and 23 were analyzed using the GLIMMIX procedure in SAS (v9.4). Behavior data was analyzed independent of challenge for the effects of age, breed, and their interaction. Means were separated using Tukey’s post-hoc adjusted LSMeans and differences between measures were detected using PDIFF. Aggression and allopreening behaviors had very low frequencies and there was insufficient data for statistical analysis, so aggression and allopreening results are reported as mean numerical observations. Data were considered significant at a *p* ≤ 0.05 and a tendency at *p* ≤ 0.10.

## Results

### Qualitative Bacteriology for *Salmonella* spp. Presence/Absence

To ensure that the chicken colony was free of exogenous *Salmonella enterica* contamination, birds were screened for the presence of *Salmonella* prior to inoculation. On day 0, all birds were negative for *Salmonella* spp. growth, while 5 (33%) CONV and 3 (20%) SG birds were positive for non-*Salmonella* spp. bacterial growth on BSA plates. One day prior to *Salmonella* challenge (day 13), 22 (92%) CONV and 22 (92%) SG birds were positive for non-*Salmonella* spp. bacteria, and all birds were negative for *Salmonella* spp. growth on BSA and BSA + NAL plates.

### Mortality and Body Weight

Mortality before moving birds into ABSL2 isolators on day 7 (*n* = 312 birds) was 6 CONV and 1 SG. Mortality after day 7 was 2 CONV-C birds between day 19 and 24.

As anticipated, the most obvious divergence was between CONV and SG birds as they aged. Breed had an effect on BW as the birds aged (Table 2). CONV tended to have a greater (*p* = 0.09) BW than SG by 24 g on day 13. On day 17, CONV tended to have a greater BW (*p* = 0.06) than SG by 45 g. CONV birds, regardless of bacterial challenge, had a greater (*p* = 0.03) BW by 70 g on day 21 and by 140 g (*p* = 0.007) on day 24 than SG. Conversely, *Salmonella* challenge did not affect BW in either CONV or SG chickens.

**TABLE 2:** Body weight (BW, g) on days 7, 13, 17, 21, and 24.

	CONV		SG			*P*-value^a^		
Age (day)	C	ST	C	ST	SEM	B	C	B*C
7	129	-	127	-	4.0	0.69	-	-
13	279	316	271	276	9.8	0.08	0.12	0.23
17	441	461	399	413	22.7	0.06	0.45	0.91
21	682^a^	709^a^	625^b^	626^b^	31.3	0.04	0.66	0.69
24	906^a^	876^a^	754^b^	747^b^	47.0	0.007	0.70	0.80

Data shown as mean BW (±SEM) of male broilers from conventional (CONV) and slow-growing (SG) breeds challenged with Salmonella Typhimurium (ST) or TSB (C) on day 14 of age.

a B, breed; C, challenge; B*C, breed*challenge interaction.

^ab^Means sharing the same letters across each row are significantly different for the main effect of breed.

### Immune Response

It was hypothesized that differences in growth rate may lead to differential immune responses or susceptibility to bacterial infection. To query the immune response to *Salmonella* spp. infection, the IgA response to infection was measured (Table 3). On the day prior to challenge (day 13), plasma IgA concentrations in C birds was 20 μg/ml greater (*p* = 0.009) than ST. As anticipated, an IgA response was mounted 1 week after bacterial challenge on day 21 where the infected chickens (ST) had greater plasma IgA levels than uninfected (C) counterparts. Plasma IgA was greater (*p* = 0.03) for ST birds at day 21 than C by 44 μg/ml (Table 3). At 10 days after infection (day 24) CONV bird IgA was greater (*p* = 0.01) than SG by 42 μg/ml (Table 3).

**TABLE 3:** Plasma IgA and IgG concentrations (μg/ml) on days 7, 13, 17, 21, and 24.

	CONV		SG			*P*-value^a^		
Age (day)	C	ST	C	ST	SEM	B	C	B*C
IgA (μg/ml)								
7	47	-	47	-	5	0.93	-	-
13	74^a^	59^b^	69^a^	44^b^	7	0.13	0.009	0.47
17	61	64	51	61	7	0.41	0.37	0.64
21	138^b^	168^a^	107^b^	164^a^	19	0.36	0.03	0.49
24	121^a^	115^a^	72^b^	88^b^	14	0.01	0.73	0.40
IgG (μg/ml)								
7	2693^b^	-	3037^a^	-	120	0.05	-	-
13^3^	1991	1709	1589	1578	189	0.19	0.44	0.47
17	1341	1218	1281	962	157	0.33	0.18	0.54
21	2120^a^	1597^a^	985^b^	1356^b^	284	0.03	0.79	0.13
24	3051^a^	2675^a^	1199^b^	1580^b^	334	0.0003	0.99	0.27

Data shown as mean concentrations (±SEM) of immunoglobulin in the plasma of male broilers from conventional (CONV) and slow-growing (SG) breeds challenged with S. Typhimurium (ST) or TSB (C) on day 14 of age.

a B, breed; C, challenge; B*C, breed*challenge interaction.

^ab^Means sharing the same letters across each row are significantly different.

**FIGURE 1:**
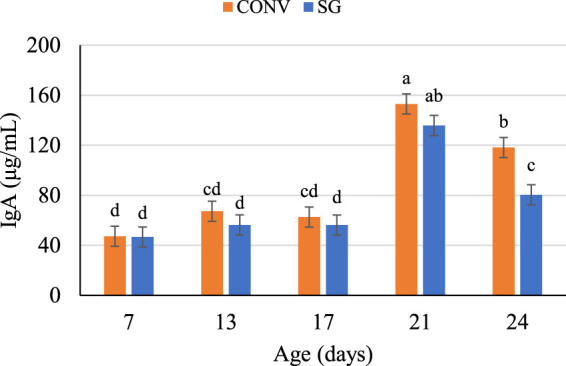
Plasma IgA concentrations (μg/ml) on days 7, 13, 17, 21, and 24. Data shown as mean IgA concentrations (±SEM) of male broilers from conventional (CONV) and slow-growing (SG) breeds challenged with *Salmonella* Typhimurium (ST) or TSB (C) on day 14 of age. ^abcd^Columns within each age not sharing the same letters are significantly different.

Independent of breed and challenge, IgA concentrations were similar between day 7 (47 μg/ml), day 13 (62 μg/ml), and day 17 (59 μg/ml), then increased (*p* < 0.0001) to 144 μg/ml on day 21 and decreased on day 24 (Figure 1). On day 24, CONV bird plasma IgA was 38 μg/ml greater (*p* = 0.01) than SG (Figure 1).

To assess the potential IgG response to *Salmonella* challenge, IgG was measured throughout the infection (Table 3). The initial observation was detection of elevated (maternal) IgG at day 7 that waned the day before challenge (day 13). On day 7, CONV birds had lower (*p* = 0.05) plasma IgG than SG by 344 μg/ml. Overall, the effect of breed on plasma IgG concentrations was significant on days 21 and 24 with the SG birds consistently demonstrating reduced plasma IgG. Day 21 and 24, CONV birds had greater (*p ≤* 0.03) plasma IgG than SG by 688 μg/ml and 1,473 μg/ml, respectively (Table 3). However, *Salmonella* challenge did not induce an increase in plasma IgG between C and ST groups at any age.

**FIGURE 2:**
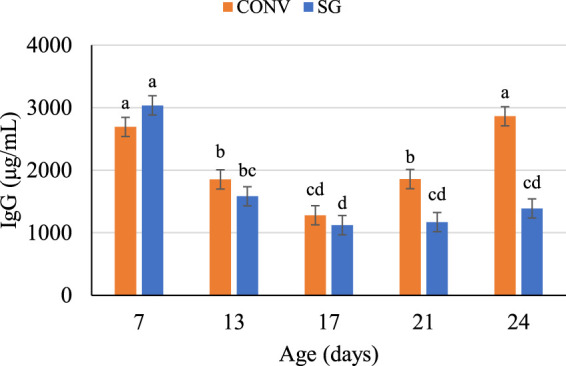
Plasma IgG concentrations (μg/ml) on days 7, 13, 17, 21, and 24. Data shown as mean (±SEM) IgG concentrations of male broilers from conventional (CONV) and slow-growing (SG) breeds challenged with *Salmonella* Typhimurium (ST) or TSB (C) on day 14 of age. ^abcd^Columns not sharing the same letters are significantly different.

There was no effect of challenge on IgG concentration across age. Independent of challenge, the effects of breed, age, and their interaction were significant (Figure 2). The plasma IgG of both CONV and SG birds were similar and decreased (*p* < 0.0001) from day 7 through 17, after which CONV and SG plasma IgG concentrations diverged (*p* < 0.0001) (Figure 2). CONV plasma IgG increased to 1,859 μg/ml on day 21 and 2,863 μg/ml on day 24, while SG plasma IgG remained lower (*p* < 0.0001) than CONV at concentrations of 1,170 μg/ml on day 21 and 1,389 μg/ml at day 24 (Figure 2).

### Gut Morphology

**FIGURE 3:**
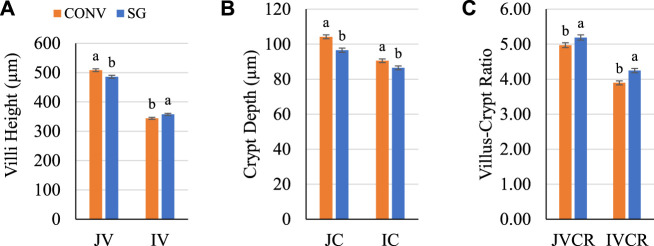
Day 7 **(A)** villus height, **(B)** crypt depth, and **(C)** VCR. Data shown as mean (±SEM) jejunum villi height (JV; μm), jejunum crypt depth (JC; μm), jejunum villus-crypt ratio (JVCR), ileum villi height (IV; μm), ileum crypt depth (IC; μm), and ileum villus-crypt ratio (IVCR) of male broilers from conventional (CONV) and slow-growing (SG) breeds when challenged with *Salmonella* typhimurium (ST) or TSB (C) on day 14 of age. ^ab^Columns not sharing the same letters are significantly different.

Gut anatomy prior to infection was assessed for differences between breeds (Figure 3). On day 7, all histological measures were significant (*p ≤* 0.04) for the main effect of breed. CONV jejunum villus (JV) height and jejunum crypt (JC) depth were both greater (*p* ≤ 0.001) than SG by 22 and 7 μm, respectively (Figures 3A,B). CONV ileum villus (IV) height was shorter (*p =* 0.008) than SG by 14 μm (Figure 3A); however, CONV ileum crypt (IC) depth was greater (*p* = 0.007) than SG by 4 μm (Figure 3B). The jejunum villus-crypt ratio (JVCR) of CONV was lower (*p* = 0.003) than SG by 0.2, and CONV ileum villus-crypt ratio (IVCR) was lower (*p* < 0.0001) than SG by 0.4 (Figure 3C).

**FIGURE 4:**
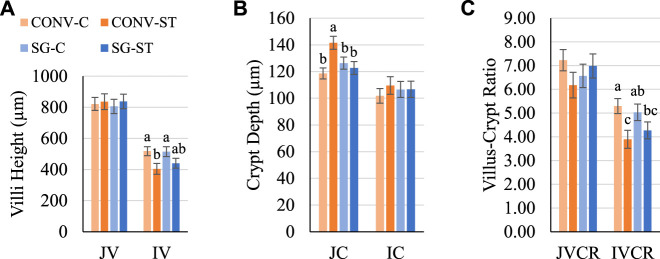
Day 24 **(A)** villus height, **(B)** crypt depth, and **(C)** VCR. Data shown as mean (±SEM) jejunum villi height (JV; μm), jejunum crypt depth (JC; μm), jejunum villus-crypt ratio (JVCR), ileum villi height (IV; μm), ileum crypt depth (IC; μm), and ileum villus-crypt ratio (IVCR) of male broilers from conventional (CONV) and slow-growing (SG) breeds challenged with *Salmonella* Typhimurium (ST) or TSB (C) on day 14 of age. ^abc^Columns within each age not sharing the same letters are significantly different.

To determine if these differences in gut anatomy persisted as the birds grew and to identify potential differential responses to *Salmonella* infection, jejunal and ileal villi anatomy were assessed again. On day 24, the main effect of challenge was significant for IV height but not JV height (Figure 4). C IV height was greater (*p =* 0.009) than ST IV by 94 μm (Figure 4A). CONV-C IV height was 114 μm greater (*p =* 0.009) than CONV-ST (Figure 4A). There was no effect of breed within challenge group. The effect of challenge was also significant for JC depth among CONV birds but not SG on day 24 (Figure 4B). CONV-C JC depth was greater (*p* = 0.05) than CONV-ST JC by 23 μm (Figure 4B). The effect of challenge on IVCR was significant on day 24 in which C IVCR was greater (*p* = 0.007) than ST IVCR by 1.1 (Figure 4C). CONV-C IVCR was 1.4 greater (*p = 0.*007) than CONV-ST on day 24.

### Correlations

To collate the data from Figures 1–4 and Tables 2, 3, correlation analyses to identify potential correlations between metrics were performed (Table 4). Although weak in strength, plasma IgA concentrations were correlated with several measures. Independent of breed, there were positive correlations between IgA and BW (r = 0.43; *p* ≤ 0.01), JV (r = 0.41; *p* ≤ 0.01), JVCR (r = 0.35; *p* ≤ 0.01), and IV (r = 0.32; *p* ≤ 0.01). Independent of breed, IgG did not correlate with any measure. CONV plasma IgA was positively correlated with BW (r = 0.46; *p* ≤ 0.01), JV (r = 0.35; *p* ≤ 0.05), JVCR (r = 0.33; *p* ≤ 0.05), IV (r = 0.31; *p* ≤ 0.05), and IgG (r = 0.21; *p* ≤ 0.05). Within SG, plasma IgA positively correlated with BW (r = 0.34; *p* ≤ 0.01), JV (r = 0.45: *p* ≤ 0.01), and IC (r = 0.43; *p* ≤ 0.05). SG plasma IgG, negatively correlated with BW (r = -0.38; *p* ≤ 0.01), IV (r = −0.54; *p* ≤ 0.01), and IVCR (r = -0.42; *p* ≤ 0.05).

**TABLE 4:** Correlations (r) between plasma IgA and IgG concentrations (µg/ml) and body weight (BW; g), jejunum villus height (μm), jejunum crypt depth (μm), jejunum villus-crypt ratio, ileum villus height (μm), ileum crypt depth (μm), ileum villus-crypt ratio, and plasma IgA and IgG concentrations of male broilers from conventional (CONV) and slow-growing (SG) breeds on day 7 and 24 when challenged with *Salmonella* Typhimurium (ST) or TSB (C) on day 14 of age.

	BW^a^	JV	JC	JVCR	IV	IC	IVCR	IgA	IgG
Both breeds									
IgA	0.43**	0.41**	0.21	0.35**	0.32**	0.28	0.12	1.00**	0.05
IgG	-0.08	-0.14	-0.02	-0.12	-0.14	0.01	-0.15	0.05	1.00**
CONV									
IgA	0.46**	0.35*	0.17	0.33*	0.31*	0.14	0.23	1.00**	0.21*
IgG	0.10	0.06	0.12	0.01	0.10	0.18	-0.01	0.21*	1.00**
SG									
IgA	0.34**	0.45**	0.22	0.33	0.32	0.43*	-0.06	1.00**	-0.17
IgG	-0.38**	-0.34	-0.15	-0.31	-0.54**	-0.17	-0.42*	-0.17	1.00**

a BW, body weight; JV, jejunum villus height; JC, jejunum crypt depth; JVCR, jejunum villus-crypt ratio; IV, ileum villus height; IC, ileum crypt depth; IVCR, ileum villus crypt ratio. *p ≤ 0.05; **p ≤ 0.01.

### Behavior

**FIGURE 5:**
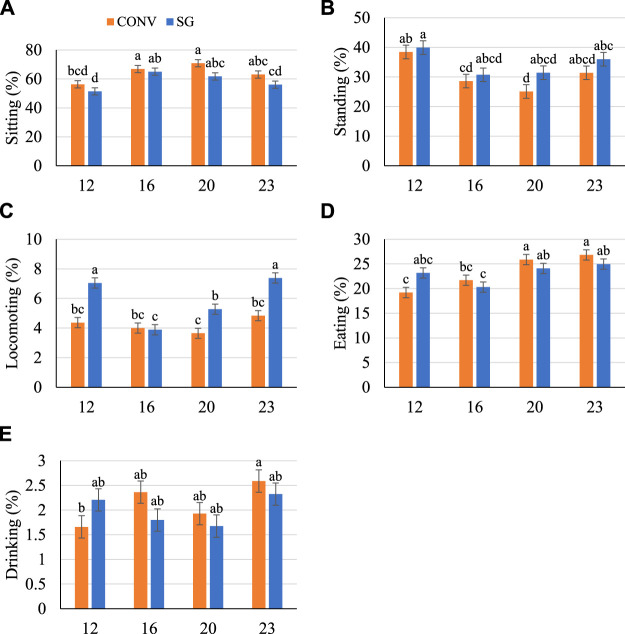
Proportion (%) of birds **(A)** sitting, **(B)** standing, **(C)** locomoting, **(D)** eating, and **(E)** drinking on days 12, 16, 20, and 23. Data shown as mean (±SEM) of male broilers from conventional (CONV) and slow-growing (SG) breeds challenged with *Salmonella* Typhimurium (ST) or TSB (C) on day 14 of age. ^abcd^Columns not sharing the same letters are significantly different.

**FIGURE 6:**
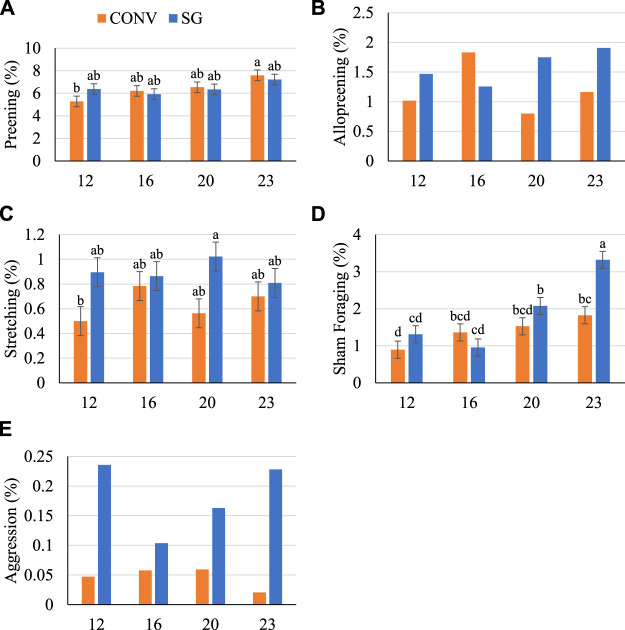
Proportion (%) of birds **(A)** preening, **(B)** allopreening, **(C)** stretching, **(D)** sham foraging, and **(E)** exhibiting aggression on days 12, 16, 20, and 23. Data shown as mean (±SEM) of male broilers from conventional (CONV) and slow-growing (SG) breeds challenged with *Salmonella* Typhimurium (ST) or TSB (C) on day 14 of age. ^abcd^Columns not sharing the same letters are significantly different.

Finally, to query for strain- or infection-associated differences in behavior, observation with quantification of common chicken behaviors was performed. There were minimal effects of challenge on the proportions of postures or behaviors exhibited in this study. Age and breed had much stronger effects and the main effects of breed, age, and their interaction are reported (Figures 5, 6).

Across all days, more CONV birds tended to sit more (*p* < 0.10) than SG (Figure 5A). There was no effect of breed on standing behavior, and both breeds generally sat more (*p <* 0.0001) and stood less (*p <* 0.0001) on days 16 and 20 compared with other days (Figures 5A,B). Fewer (*p* < 0.0001) CONV birds locomoted than SG on days 12, 20, and 23 by 2.7, 1.6, and 2.6%, respectively (Figure 5C). There was no difference between breeds in proportion of birds eating, but more (*p <* 0.0001) birds ate as they aged from 21.8% on day 12 to 25.9% on day 23 (Figure 5D). The proportion of birds drinking generally increased (*p =* 0.01) from 1.93% on day 12 to 2.46% on day 23 (Figure 5E).

There was no effect of breed on the proportion of birds preening (Figure 6A), however, a numerically greater proportion of SG birds were allopreening on days 12, 20 and 23 than CONV (Figure 6B). Overall, more (*p =* 0.007) SG stretched than CONV by 0.3% (Figure 6C). A similar proportion of birds were sham foraging on days 12 through 20 (between 1 and 2%) and a greater (*p* = 0.04) proportion of SG birds sham foraged on day 23 than CONV (Figure 6D). Despite the low proportion of birds sham foraging, at day 23 fewer (*p* < 0.05) SG-ST birds sham foraged than SG-C by 1.6% (data not shown). Additionally, very low proportions of birds were observed exhibiting aggression (less than 0.5%), but a numerically greater numerical proportion of SG birds exhibited aggression than CONV at every age (Figure 6E).

## Discussion

The selection for rapid growth in modern broilers has resulted in global animal welfare concerns and consideration towards the use of slower-growing breeds. Additionally, *Salmonella enterica* remains an ever-present threat to both the broiler industry and human health. Health and welfare are very interrelated, but these relationships may not always be direct. As such, the differences in physiological and behavioral indicators of health and welfare in two breeds of broiler chickens varying in growth rate when challenged with *Salmonella* Typhimurium were evaluated.

As expected, *Salmonella* Typhimurium challenge did not impact morbidity, mortality, or body weight, and conventional broilers weighed more than slow-growing by day 13. However, both breeds had lower body weight and body weight gain than expected (Aviagen, 2019), which may be attributed to the feed provided in this study. The feed used in this study was a commercial backyard chicken feed, which was not specifically formulated for each dietary phase commonly provided to commercial broilers. In a study by Torrey et al. (2021) evaluating differences in 16 broiler breeds varying in growth rate, all birds were provided a diet formulated for a moderate slow-growing breed and, similar to our study, reduced growth occurred among the conventional breeds. Given the relationship between gut morphology and absorptive function of the intestines (Yamauchi et al., 2010), this may have also caused the birds in this study to have shorter villi and crypt measures than broilers of the same age or younger in other studies (Fasina et al., 2010; Lee et al., 2010; Golder et al., 2011).

The conventional and slow-growing breeds differed in their immunoglobulin response. Antibody concentrations increase with age in the chicken (Parmentier et al., 2004), and adult chickens are expected to have plasma IgA concentrations of 600 μg/ml and plasma IgG (IgY) concentrations of 4,500–5,000 μg/ml (Tizard, 2002). The plasma immunoglobulin concentrations in this study at 24 days of age appear consistent with these findings, given that broilers are not fully mature at this age.

Avian IgA is the most predominant immunoglobulin in intestinal secretions, protecting intestinal surfaces from pathogen invasion (Tizard, 2002). Both breeds had elevated IgA responses to challenge on day 21 (1-week post-challenge) but the conventional breed had greater IgA concentrations than the slow-growing breed at day 24. Peaks in antibody levels such as IgA are generally associated with pathogen clearance (van der Most et al., 2010). If this is the case, the responses indicate the slow-growing breed may have a more effective IgA response to *S.* Typhimurium challenge than the conventional breed. However, a strong humoral immune response may not necessarily be effective or indicative of strong immune function (Barrow et al., 2012).

Within the control group of both breeds, baseline IgA concentrations were slightly elevated at day 13, which was 24 h prior to challenge. Individual variation of randomly sampled control birds could have contributed to this difference, but time of day at sampling could have also caused elevated IgA concentrations. Melatonin is known to have a role in circadian rhythm but also modulates the immune system in mammals, capable of influencing antibody production (Cernysiov et al., 2009). In laying hens administered melatonin intraperitoneally at 70 weeks of age, plasma IgA, IgG, and IgM levels were elevated (Hao et al., 2020). Circulating melatonin levels in the chicken increase during dark hours and melatonin production decreases with light stimulation (Pelham, 1975). In the present study, sampling occurred in the morning with all control-assigned birds sampled before the challenge-assigned birds. Thus, control birds were sampled earlier in the day and these birds could have had greater concentrations of melatonin, leading to greater plasma IgA levels than the later-sampled challenge birds. However, this pattern was not observed on any other day. Plasma IgA concentrations peaked in both challenge and control treatments on day 21 but were greater in challenge birds than in control birds. Located beneath intestinal mucosal surfaces, B-cells produce IgA in the intestines, of which some ends up in the bloodstream (Tizard, 2002). Thus, elevated plasma IgA levels in the bloodstream can reflect a response to *S.* Typhimurium infection in the intestines of challenged birds.

Another vital component of the chicken immune response to infection is IgG (Gharaibeh and Mahmoud, 2013). At day 7, IgG concentrations were greater in slow-growing birds than conventional, which may reflect a greater natural level of maternal antibodies in the blood. IgG, also termed IgY (immunoglobulin of the yolk), is a maternal antibody transferred to chicks through the egg yolk and lasts up to 10 days post-hatch in the chick, providing critical early life immune protection to chicks until immunocompetence is attained (Carlander et al., 1999; Gharaibeh and Mahmoud, 2013). Greater levels of maternal antibodies can reflect stronger early life immune protection (Marcq et al., 2011) and maternal IgG is especially important in broiler health due to the short lifespan of broilers in commercial production systems (Gharaibeh and Mahmoud, 2013). Broiler secondary immune organs, such as the spleen and cecal tonsils, and the resulting humoral response are not mature enough to mount an immune response until approximately 12 days of age (Mast and Goddeeris, 1999). Furthermore, the broiler immune response is not fully developed until approximately 30 days of age (Song et al., 2021). In the present study, maternal antibodies may have persisted at elevated concentrations in the slow-growing breed up to day 7 than in the conventional breed, indicating the slow-growing breed may have stronger and longer-lasting early life immune protection.

There was no apparent plasma IgG response to *S.* Typhimurium challenge in either breed. The present study was limited to the use of commercial ELISA kits to measure plasma immunoglobulin response. Thus, all plasma IgA and IgG results in the current study reflected total plasma concentrations of either immunoglobulin but not *S.* Typhimurium antigen-specific immunoglobulins, and actual immunoglobulin responses to the challenge may have been masked by baseline circulating levels of immunoglobulin. This is especially the case for IgG due the naturally high baseline levels of circulating IgG in the chicken (Tizard, 2002).

IgG concentrations increased after day 17 in the conventional breed but not the slow-growing breed. The selection for increased growth rate in broilers may have resulted in increased lymphoid organ development or earlier immune system maturation in the conventional breed. However, the rate of development or heightened antibody levels do not necessarily correspond with a stronger or more efficient immune response (van der Most et al., 2010; Barrow et al., 2012). Further research is needed to investigate the relationship between growth rate and the development of immunocompetence in broilers.

The conventional and slow-growing breeds differed in both ileum and jejunum histomorphological measures at day 7. The conventional breed had greater jejunal villus height and crypt depth than the slow-growing breed, which may suggest greater absorptive efficiency relative to the birds from this breed’s enhanced growth rate. On the other hand, the slow-growing breed had greater ileum villus height but shallower ileum crypts than the conventional breed. Growth-related differences in gut morphological measures, particularly villus height, have been documented, noting that selection for growth in broilers has resulted in longer villi when compared to White Leghorns (Yamauchi and Isshiki, 1991). Between both intestinal segments, however, the slow-growing breed had greater day 7 villus height to crypt depth ratios than the conventional breed, which could indicate reduced cellular turnover in the jejunum and ileum (Seyyedin and Nazem, 2017). Gut morphology measures from the intermediate days would have provided further insight on the development of intestinal structure within each breed as the birds aged.

The breed intestinal histomorphological differences at day 7 disappeared by day 24 (10 days post-challenge). However, challenge impacted gut morphology, especially in the conventional breed. Conventional birds challenged with *S.* Typhimurium had deeper jejunum crypts than controls and slow-growing birds, which may indicate increased enterocyte production in the crypts of conventional birds to compensate for cellular loss at the villi tips due to infection Fernando and McCraw (1973). Fernando and McCraw (1973) found that 14-week-old male White Leghorn chicks infected with *Eimeria acervulina* had reduced jejunal villus heights and increased crypt depths, which then returned to normal or greater lengths and depths 5–6 days post-infection. However, challenge did not appear to impact jejunum villus height in either breed in the present study. Others have noted no significant differences in any gut morphology measures in broilers challenged with *Salmonella* Enteritidis (Gomes et al., 2014).

Correlations were only reported for the relationships between antibody concentrations and all other measures, as the relationship between gut morphology and body weight is well-established (Yamauchi and Isshiki, 1991; Yamauchi et al., 2010). There was a positive correlation between IgA concentrations and body weight among both breeds, which was slightly stronger in the conventional breed. Additionally, the correlation between IgG and body weight was negative in the slow-growing breed, but a very weak and insignificant positive correlation was detected in the conventional breed. IgA and IgG concentrations positively correlated with one another in the conventional breed, but not the slow-growing breed. Combined, these support the possibility that selection for increased growth rate in the conventional breed may unintentionally select for earlier development of immunocompetence. Plasma IgG concentrations had little to no significant relation to gut morphological measures, except in the slow-growing breed where IgG and ileum villus height (and ileum villus height to crypt depth ratio) negatively correlated. On the other hand, plasma IgA concentration positively correlated with multiple gut morphology measures, such as jejunum villus height. This may reflect a relationship between intestinal morphology and intestinal humoral secretions, as plasma IgA concentration can be representative of intestinal IgA concentration (Tizard, 2002).

Few behavioral differences between breeds were found in this study, and there was a minimal effect of challenge on behavior. When comparing the first (day 12) to the last (day 23) of video recording days, more birds sat and fewer stood as they aged. However, the proportion of birds locomoting was similar between the first and last day. Generally, locomotion-type behaviors, such as walking or running, also become less frequent as broilers age (Bokkers and Koene, 2003; Dixon, 2020). The birds in this study were housed in biosafety isolators with space allowance and stocking density according to the Ag Guide (FASS. Federation of Animal Science Societies, 2010), but isolator space still restricted opportunities for movement. In a study involving male Peterson Arbor Acre broilers, Pelham (1975) found that broilers housed in smaller pens utilized less total space than broilers housed in larger pens, likely due to reduced movement opportunities. It is possible that space limitations affected the frequency at which locomotion occurred and was observed. Generally, slow-growing birds sat less and locomoted more than birds from the conventional breed in the current study. These differences in locomotive activity between breeds that vary in growth rate have been reported in previous research (Bokkers and Koene, 2003; Wallenbeck et al., 2016; Dixon, 2020; Yan et al., 2021).

A unique finding in this study was more birds sat and fewer stood and locomoted following the oral gavage (days 16 and 20), independent of both breed and challenge. We hypothesize that this was a behavioral indicator of stress following gavage at day 14. The use of a gavage to deliver a substance orally is an invasive and stressful event and has been evidenced by reports of increased plasma corticosterone of rats orally gavaged with corn oil (Brown et al., 2000). Stress may cause long-term behavioral consequences, resulting in reduced activity manifested through increased sitting. For example, Ross broiler chickens that underwent heat stress at 32°C for 10 h daily from 15 to 43 days of age displayed increased sitting and reduced walking and standing (Wang et al., 2018). The inclusion of a negative (no gavage) control group would have been beneficial to this study to determine if the gavage process caused stress because it is unknown to what extent the gavage causes stress in chickens and research is needed to further investigate these effects.

The proportion of birds sham foraging generally increased with age, and it was observed that this was more pronounced among slow-growing birds. The slow-growing breed sham foraged more on each successive day, except day 16. Breed differences in foraging behavior are more obvious between broilers and layers. When a hybrid broiler breed and layer breed were provided free access to feed and to feed mixed in wood shavings, the broilers showed less foraging and greater inactivity than the layers (Lindqvist et al., 2006). Foraging behavior has been reported to vary between broiler genotypes, with a tendency for slower-growing broiler breeds to forage or engage in exploratory behaviors more than medium- (Almeida et al., 2012) and conventional breeds (Yan et al., 2021). However, other studies have reported no differences between conventional and slow-growing broiler foraging behavior (Wallenbeck et al., 2016).

The slow-growing broilers exhibited more social and agonistic behaviors than conventional in the current study. For example, more slow-growing birds engaged in allopreening (except on day 16) and aggression than conventional. Increases in aggression and displacement preening (preening that results from frustration) can indicate environment-related frustration in confined chickens (Calder and Albright, 2021), which may indicate the slow-growing breed was more affected by the housing environment or other factors in the present study. However, the observed proportions of these behaviors across all ages were very small (less than 2%) and a larger study involving more birds may have resulted in more representative time budgets. Domesticated farm animals, such as broilers, are less prone to respond behaviorally to sickness (Berghman, 2016). In this study, *S*. Typhimurium challenge did not induce sickness behaviors, such as a ruffled feathers or hunched posture, as neither behavior was observed. All results of this study were impacted by the limited study length of 24 days. Measures taken beyond the end of the present study through market age would have provided greater clarity as to breed related differences and the response to *S.* Typhimurium infection regarding body weight, immune response, gut morphology, and behavior. Further research is needed to investigate differences between conventional and slow-growing broiler breeds to determine the effect of *S*. Typhimurium infection on the frequency of social and agonistic behaviors.

The results of this study indicate that meaningful genotypic and phenotypic differences exist in conventional broilers compared to slow-growing broilers with regards to body weight, immune response, gut morphology, and behavior when challenged with *Salmonella* Typhimurium. In the present study, broilers from the conventional breed were heavier, had greater jejunum villus height with lower crypt depth, and had earlier increased IgG concentrations on days 21 and 24 of age, which may indicate earlier or faster immune development than the slow-growing breed. The slow-growing birds appeared to be more resilient to *Salmonella* challenge in that their jejunum crypt depth was unaffected by challenge, and they had greater plasma concentrations of maternal IgG, indicating greater early life immune protection. The slow-growing breed sat less and engaged in more sham foraging, allopreening, and aggression behaviors than the conventional breed and *S.* Typhimurium challenge reduced sham foraging in the slow-growing breed, but not the conventional breed. The *S.* Typhimurium challenge impaired intestinal morphology 10 days post-challenge and elevated IgA concentrations 7 days post-challenge in both breeds. Delineating the differences in basal and *Salmonella*-challenged phenotypes of broilers with divergent growth rates provides useful information for genetic, nutritional, and management decisions. Further research is needed to understand the extent of the differences between conventional and slow-growing broiler immune function, gut development, sickness behavior, and resistance to foodborne pathogens.

## Data Availability

The raw data supporting the conclusion of this article will be made available by the authors, without undue reservation.
